# IgG4-Related Periaortitis Initially Suspected of Being an Aortic Intramural Hematoma in the Ascending Aorta

**DOI:** 10.3400/avd.cr.21-00024

**Published:** 2021-12-25

**Authors:** Kazuki Takahashi, Shinsuke Kikuchi, Keisuke Kamada, Ai Tochikubo, Daiki Uchida, Atsuhiro Koya, Hiroyuki Kamiya, Nobuyoshi Azuma

**Affiliations:** 1 Department of Vascular Surgery, Asahikawa Medical University, Asahikawa, Hokkaido, Japan; 2 Department of Cardiac Surgery, Asahikawa Medical University, Asahikawa, Hokkaido, Japan

**Keywords:** IgG4-related disease, aortitis, intramural hematoma

## Abstract

Immunoglobulin G4-related disease (IgG4-RD) can affect various organs, including the cardiovascular system. In this study, we described the case of a 72-year-old man with periaortitis both in the ascending and terminal aorta related to IgG4-RD. He presented with swelling in the left leg. Computed tomography (CT) showed increased wall thickness of the ascending aorta and retroperitoneal fibrosis, which, in turn, caused deep vein thrombosis. Using positron emission tomography-computed tomography, the patient was diagnosed with IgG4-RD in the aorta. Although it was difficult to distinguish intramural hematoma (IMH) from IgG4-related periaortitis, treatment with steroids has dramatically improved his periaortitis. IgG4-related periaortitis should be differentiated from IMH due to their similar morphologies.

## Introduction

Immunoglobulin G4-related disease (IgG4-RD) is defined as a systemic inflammatory disease characterized by elevated serum levels of IgG4, infiltration of IgG4-positive lymphocytes, and storiform fibrosis.^1)^ Several reported cases of retroperitoneal fibrosis (RF) associated with IgG4-RD responded dramatically to steroid therapy.^2)^ IgG4-RD can affect any organ in the body, and IgG4-RD in the cardiovascular system has been previously reported.^3,4)^ However, there are very few reports of IgG4-related periaortitis mimicking an intramural hematoma (IMH). These two conditions present with radiologic similarities, leading to misdiagnosis and treatment.^5,6)^ Herein, we describe the case of IgG4-related periaortitis in the ascending aorta with RF and the development of acute deep vein thrombosis (DVT).

## Case Report

**Figure 1:**
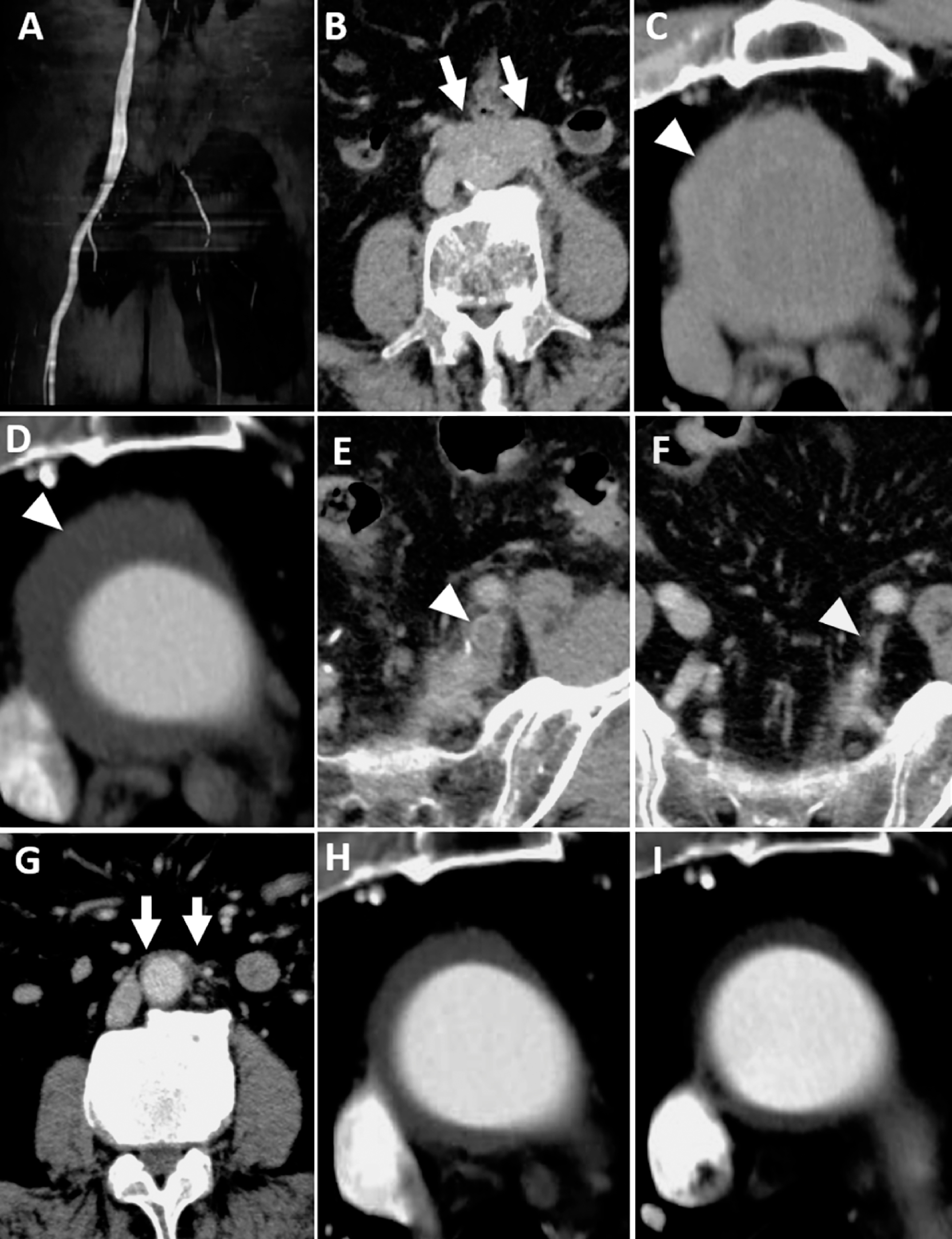
Fig. 1 Magnetic resonance angiography (MRA) and computed tomography (CT) findings. MRA shows occlusion of the left iliac vein (**A**). Plain CT shows a mass (arrows) around the terminal aorta (**B**). Plain CT (**C**) and enhanced CT (**D**) show increased wall thickness of the ascending aorta (arrowheads) before starting the steroid treatment. Also depicted are the left iliac vein thrombosis (arrowhead) (**E**), the left iliac vein was shrunk (arrowhead) (**F**), and a mass (arrows) around the terminal aorta (**G**) at 4 months after anticoagulant therapy and steroid treatment. Wall thickness of the ascending aorta is shown at 4 months (**H**) and 12 months (**I**) after steroid treatment.

**Figure 2:**
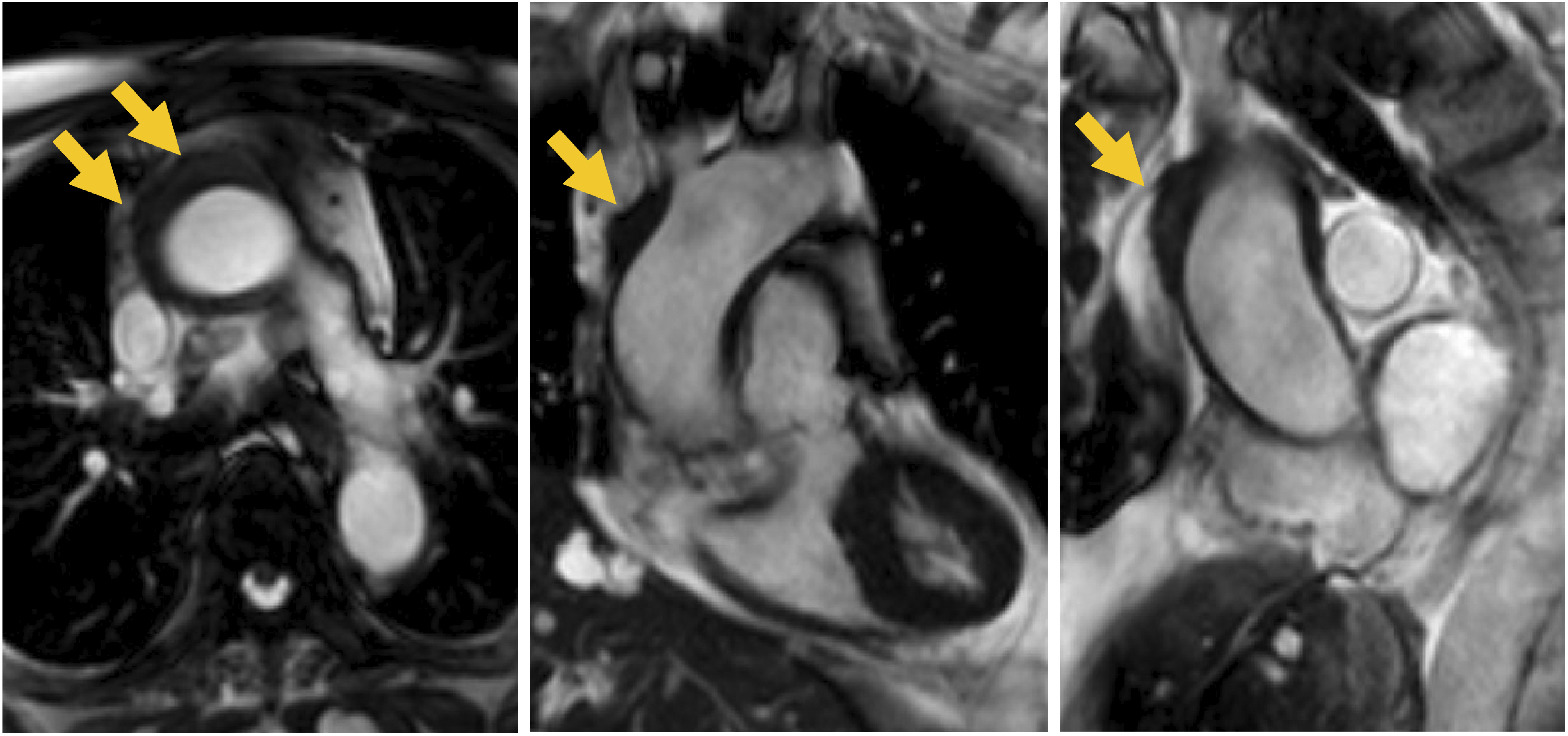
Fig. 2 T2-Weighted magnetic resonance imaging shows thickening of soft tissue around the ascending aorta.

**Figure 3:**
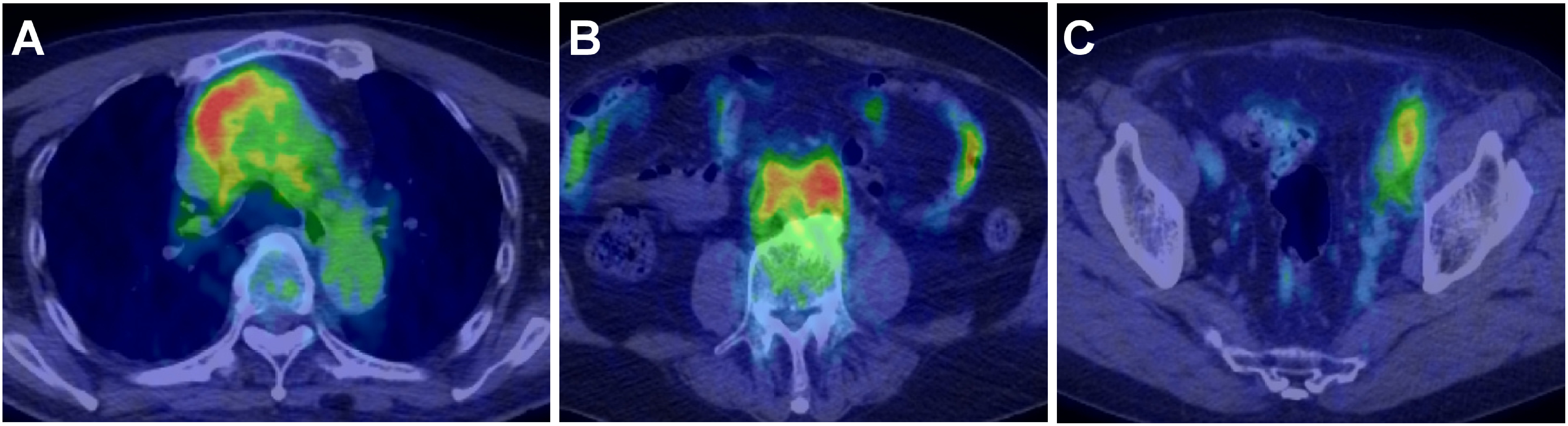
Fig. 3 18F-fluorodexyglucose positron emission tomography (18-FDG-PET). Enhanced uptake of 18F-FDG was observed (**A**) around the ascending aorta (standardized uptake value (SUV) max 6.4), (**B**) the retroperitoneal regions (SUV max 6.2), and (**C**) the left iliac vein region (SUV max 3.9).

A 72-year-old man with a history of RF presented with swelling and pain of the left lower extremity. The symptoms were attributed to acute DVT diagnosed by magnetic resonance angiography (MRA), which showed occlusion of the left iliac vein (**Fig. 1A**). As per his laboratory data, an increase in C-reactive protein (CRP, 3.37 mg/dL) and D-dimer (3.50 µg/mL) was noted. Plain computed tomography (CT) demonstrated a mass around the terminal aorta that was likely related to RF (**Fig. 1B**). Surprisingly, an abnormal finding in the ascending aorta, suggestive of aortic dissection, was also noted (**Fig. 1C**). Enhanced CT was immediately performed to diagnose the acute aortic dissection. The CT revealed a lesion suggestive of IMH in the ascending aorta, with a maximum dimension of approximately 5.3 cm (**Fig. 1D**). This abnormality was consistent with a circular IMH with a thickness of 15 mm, but no entry tear or intimal flap was determined. Additionally, the patient’s vital signs were normal, and he never experienced any episodes related to the onset of aortic dissection, such as chest pain or back pain. Ultrasound sonography did not identify any abnormal intimal flap or aortic valve function. We finally decided to observe the patient for abnormal findings in the ascending aorta with no surgical intervention. This decision was based on the following factors: lack of chest symptoms and typical findings of aortic dissection, a history of RF, and the possibility of vasculitis. Regarding the treatment of DVT, invasive therapy, including transcatheter thrombolysis and aspiration thrombectomy, was avoided due to unexpected aortic abnormalities. Instead, intravenous anticoagulant and thrombolysis using urokinase (240,000 units/day) and heparin (10,000 units/day) were started to treat the DVT. These treatments were then switched to oral apixaban on the third day. Laboratory examination showed an elevated erythrocyte sedimentation rate (ESR, 69 mm/h) and a serum IgG4 level of 279 mg/dL (normal range, 4–110 mg/dL). Magnetic resonance imaging (MRI) showed a soft mass around the ascending aorta (**Fig. 2**). F-18 fluorodeoxyglucose (FDG) positron emission tomography-CT (PET-CT) indicated heterogeneous FDG uptake around the left common iliac vein (standardized uptake value (SUV) max 3.9), retroperitonea (SUV max 6.2), and ascending aorta (SUV max 6.4) (**Fig. 3**). As a result, IgG4-related vasculitis was suspected as the cause of these abnormal findings. Unfortunately, no pathological finding was obtained. Thus, IgG4-RD was potentially diagnosed using the algorithm of the comprehensive diagnostic criteria for IgG4-RD.^3)^ The patient was prescribed 30 mg/day prednisolone (0.34 mg/kg/day) for 4 weeks. After that, the dosage was gradually reduced. The left lower extremity diameter, CRP, and serum IgG4 were noted to improve after the start of prednisolone. The left iliac vein with enhanced uptake of 18F-FDG was shrunk, and thrombi remained despite anticoagulant therapy (**Fig. 1E**). Imaging showed that the segment of the iliac vein with enhanced uptake of 18F-FDG had shrunk, suggesting that left lower extremity edema improvement may depend on the improvement of RF after steroid therapy (**Figs. 1F** and **1G**). Three months later, the serum IgG4 level was normalized (67 mg/dL) and remained normal for 12 months. Intriguingly, the periaortic tissue around the ascending aorta also markedly decreased (**Figs. 1H** and **1I**). However, when the dose of prednisolone was reduced to 5.0 mg, IgG4 level increased (108 mg/dL), and the thickness of the periaortic tissue around the ascending aorta increased. Currently, the dose of prednisolone is increased, and the patient is being followed up. Thus, his condition is regarded as a possible diagnosis of IgG4-RD, indicating that the RF, subsequent DVT, and periaortic abnormalities were probably caused by IgG4-RD.

## Discussion

IgG4-RD was first recognized in autoimmune pancreatitis in 2001, and IgG4-related vascular disease was first reported in 2008.^3)^ In terms of IgG4-related aortitis, diagnostic certainty is difficult in many cases due to its radiologic similarities between aortitis, IMH, and aortic dissection. Thus, IgG4-related aortitis can be confused with these acute aortic diseases using CT imaging in emergency situations due to the increased aortic wall thickness.^5,6)^ In this present case, emergency surgery to address the findings of the ascending aorta was avoided because of the possibility of vasculitis based on the development of RF and acute DVT. As a result, medications, including steroid therapy, have remarkably improved the aortic abnormalities and retroperitoneal lesions. Thrombolysis was limited to treating the DVT; however, the improvement of RF may have contributed to the improvement of edema in the left lower extremity.

The differential diagnosis between IMH and IgG4-related aortitis has been identified of great importance since the treatments differ significantly in terms of their invasiveness; IgG4-RD can be treated with medication, whereas treatment of IMH in the ascending aorta requires a thoracotomy. Although IMH is much less common than acute aortic dissection, they have similar mortality rates.^7)^ In two case reports, a thoracotomy was performed for IgG4-related aortitis because differentiation could not be preoperatively performed.^5,6)^ In this case, thrombolysis could have been used to manage acute DVT; however, this approach would have been contraindicated for IMH. Therefore, using the least necessary intervention and managing the case with anticoagulant therapy alone might be considered a safer strategy. To address this diagnostic hurdle, MRI can quickly distinguish between IgG4-related aortitis and IMH in an emergency room, as shown in **Fig. 2**. On MRI, acute IMH shows T2 signal hyperintensity of the hematoma; however, this case differs from IMH in that the T2-weighted image showed a low signal in the thickened wall.^8)^

IgG4-related vasculitis has no specific diagnostic criteria; it is diagnosed with the same diagnostic criteria as normal IgG4-RD. A definitive diagnosis requires tissue obtained by biopsy or surgery. The typical pathologic features include adventitial inflammation, fibrous thickening, and lymphoplasmacytic infiltration.^3)^ However, obtaining a cardiovascular biopsy remains difficult and is deemed high risk; thus, a cardiovascular biopsy cannot always be obtained. Therefore, FDG PET-CT is useful for the diagnosis and evaluation of disease activity. In addition, FDG PET-CT may indicate other biopsy sites for diagnosis. In the case of our patient, serum IgG4 was elevated, and a remarkable accumulation of FDG was observed around the ascending aorta by PET-CT. Disease activity was also confirmed in RF and the left iliac vein. Disease activity was especially evidenced by the thrombosis formation in the left iliac vein, which could be associated with vasculitis, as shown in **Fig. 3C**. However, no other biopsy site exists, and no definitive pathologic diagnosis could be made. When the current diagnostic criteria are applied, a definitive diagnosis of IgG4-RD cannot be made without a biopsy finding, but IgG4-RD should be considered.

Steroid therapy has been well recognized as the first-line therapy for IgG4-RD. Mizushima demonstrated that perivascular lesions of periaortitis improved with steroid therapy, and changes in serum IgG4 levels after steroid administration reflect the therapeutic effect.^9)^ However, in one case, rapid aortic wall thinning after steroid therapy caused the rupture of an aortic aneurysm.^10)^ Thus, patients treated with steroid therapy for suspected IgG4-related aortitis should be followed up due to the possibility of aortic aneurysm formation with steroid treatment.

## Conclusion

In this study, we presented the case of IgG4-related aortitis in the ascending aorta with RF and acute iliac vein occlusion. IgG4-related aortitis has been noted to have radiologic similarities to acute aortic diseases, such as aortic dissection and IMH. Treatment plans differ widely for patients according to the diagnosis, and we should keep this fact in mind.

## Additional Note

The patient provided written consent at the time of discharge for his information to be used for research and publication.
